# A New Family-Based Approach for Detecting Allele-Specific Expression and for Mapping Possible eQTLs

**DOI:** 10.3390/ani15182766

**Published:** 2025-09-22

**Authors:** Maher Alnajjar, Zsófia Fekete, Tibor Nagy, Zoltán Német, Agshin Sakif, Nóra Ninausz, Péter Fehér, Viktor Stéger, Endre Barta

**Affiliations:** 1 Department of Genetics and Genomics, Institute of Genetics and Biotechnology, Hungarian University of Agriculture and Life Sciences, Szent-Györgyi A. u. 4, H-2100 Gödöllő, Hungary; alnajjar.maher@uni-mate.hu (M.A.); nagy.tibor2@uni-mate.hu (T.N.); sakif.agshin@phd.uni-mate.hu (A.S.); nora.ninausz@gmail.com (N.N.); feher.peter.arpad@uni-mate.hu (P.F.); steger.viktor@uni-mate.hu (V.S.); 2 Department of Environmental and Biological Sciences, University of Eastern Finland, Yliopistokatu 2, 80100 Joensuu, Finland; zsofia.fekete@uef.fi; 3 Department of Biochemistry and Molecular Biology, Faculty of Medicine, University of Debrecen, Egyetem tér 1, H-4032 Debrecen, Hungary; 4 Department of Pathology, University of Veterinary Medicine, István u. 2, H-1078 Budapest, Hungary; nemet.zoltan@univet.hu

**Keywords:** allele-specific expression, cis-regulatory elements, transcription factor binding sites, rabbit, meat

## Abstract

Allele-specific expression (ASE) reflects the unequal expression of the parental alleles and can imply functional variants in cis-regulatory elements. The conventional ASE detection methods often depend on the presence of heterozygous variants in transcripts or sequencing a large number of individuals, both of which are often limited. In this study, we present a family-based strategy for detecting ASE and potential cis-regulatory elements utilizing both RNA-seq and whole-genome sequencing (WGS) from a pedigree. Using a rabbit family consisting of two divergent parents and their eight offspring, we identified 913 ASE genes by analyzing inheritance patterns of gene expression levels. Expression was classified into three levels—high, medium, and low—and used to define seven distinct expression groups across the family (e.g., H_L: high in the mother, low in the father, and intermediate in the offspring). Many ASE genes lacked heterozygous exonic variants, and inference was achieved via RNA read count patterns. We also pinpointed conserved transcription factor binding sites (TFBS) with sequence variants showing similar inherited genotypic patterns (e.g., AAxBB), suggesting their regulatory roles as eQTLs. Differential gene expression (DEG) analysis between the parents highlighted some candidate genes related to meat production and quality traits. Our findings show that the family-based method using RNA-seq and WGS data is promising for exploring ASE and mapping possible eQTLs.

## Simple Summary

Allele-specific expression (ASE) is a valuable tool for finding different traits on the gene expression level. However, existing research often relies on heterozygous transcribed variants or sequencing large cohorts, which can be costly and sometimes limited. To address these challenges, we developed a novel family-based approach that combines RNA sequencing and whole-genome sequencing (WGS) in a family model to detect ASE and infer cis-regulatory variants without relying on population data or heterozygous SNPs in the exonic region. We applied this method to a hybrid rabbit family consisting of two genetically distinct parents and eight offspring. We identified 913 ASE genes by estimating the inheritance patterns of gene expression levels, even in the absence of heterozygous variants in the transcriptomes. Expression levels were categorized into three states—high, medium, and low—and genes were classified into seven expression patterns. We also identified conserved transcription factor binding sites (TFBS) near genes that showed consistent genotype patterns, supporting their role as cis-acting elements. Furthermore, differential expression analysis between parents revealed genes that are potentially relevant for meat production traits.

## 1. Introduction

The first observable phenotype of all genes in living cells is the synthesis of the corresponding RNA molecules [1]. The mechanisms of gene expression regulation are very complex. There can be different transcript forms of a single gene, and which, where, when, and how many transcripts are transcribed from a gene are strictly regulated [2]. This regulation is encoded into the DNA in different ways. Specific proteins bind cis-regulatory elements, and alternative forms of these proteins with different binding affinities can exist. These proteins are also expressed from genes. Thus, gene expression is also regulated by when, which forms, and how many such proteins are present in the nucleus. Further, gene expression is also regulated by whether a given cis-regulatory element is available for binding [3,4]. Despite this complexity, gene expression can be measured by sequencing the isolated mRNA molecules. Bulk RNA-seq offers a reliable approach for studying gene expression under different conditions, thus providing a useful tool for better understanding the fundamental processes of living cells [5].

In diploid organisms, each gene encoded on the autosomes is present in two alleles, one from each parent. Therefore, if the two parents have different cis-regulatory elements or trans factors, the two copies of the gene will be expressed at different levels. In this case, we can speak of allele-specific expression (ASE), as reviewed by [6]. In principle, cis-regulatory elements influence the expression levels of nearby genes in an allele-specific manner, whereas trans-regulatory factors tend to regulate both alleles of distant genes [7]. To fully understand the influence and implications of these variants in transcription factor-binding sites (TFBS), the causal regulatory variant and the corresponding gene (the target gene) should be identified [8]. Of course, if we quantify the gene expression level using, for example, RNA-seq analysis, we can determine the sum value from the two parents’ genes. However, if the exons of a given gene have any heterozygosity, we can simply determine the ratio of the mRNAs from the two parents [9]. Although ASE theoretically must exist in every diploid organism, it is challenging to detect.

The most common ASE method utilizes heterozygous sites in transcribed exons, where RNA sequencing reads carry either the maternal or paternal allele. By counting each type and calculating its ratio, we can assess allele-specific expression. This requires heterozygosity in the given individual and that also the allele with lower expression yields at least some reads. Therefore, we can use this approach on an F1 individual of genetically distant parents or a cohort of individuals with highly expressed genes. This is a significant limitation, as this approach cannot be used to identify candidate cis-regulatory elements. Expression genome-wide association studies GWAS (eGWAS/TWAS) instead compares gene expression between phenotype-defined cohorts. In this case, statistical analysis can be applied to the gene expression values of individuals instead of the genotypes. This approach can be combined with the traditional GWAS analysis of the same cohorts [10]. The limitations of this kind of approach include its high costs and the complexity of the RNA-seq results. On the other hand, the resulting expression quantitative trait loci (eQTLs) help to pinpoint the genetic regions connected to a given phenotype. This approach is especially useful in breeding [11].

The ultimate goal of these ASE analyses is to identify variants in cis-regulatory elements that are responsible for changes in expression levels by affecting the binding of a given transcription factor. The typical approach for such analysis is to find a variant in a potential transcription factor binding site (TFBS) and associate the three potential genotypes (AA, AB, and BB) with three different expression levels (low, moderate, and high). To accomplish this, we must perform whole-genome and RNA sequencing analyses on multiple samples.

Several studies on ASE have been conducted in humans, mice, crops, and farm animals [11,12,13,14,15] mainly to link examples of ASE to specific traits and, in some cases, identify regulatory SNPs (rSNPs) underlying altered expression. However, to our knowledge, no study has attempted a comprehensive evaluation of potential ASE scenarios within a whole-family context.

The accurate discovery of heterozygous variants is required for conducting a successful ASE experiment, either in the target gene (RNA-seq) or in a TFBS (WGS). Variants called from RNA-seq carry the risk of false negatives, i.e., heterozygous sites called as homozygous, when allele expression is extremely imbalanced [6]. Conversely, genotyping errors from DNA sequencing might lead to a false positive interpretation, where homozygous variants are called as heterozygous [16].

In this study, we established a rabbit family model with eight offspring, enabling more accurate phenotyping and genotyping. This setup allowed us to examine how the expression levels were inherited across the family and how this matched the Mendelian inheritance of the overlaying variants. Many genes showed three distinct expression levels, typically matching the corresponding genotype in that region. We also identified variants in conserved transcription factor binding sites near these ASE genes, which exhibited a matching genotype pattern across the family. Therefore, they may serve as potential rSNPs that lead to altered expression levels.

## 2. Materials and Methods

### 2.1. Samples

Samples were prepared from muscle tissue (thigh and back) taken from a breeding pair of rabbits (Oryctolagus cuniculus) and their eight offspring. The mother belonged to the Hycole XXL line, which was selected for meat production due to its large body weight over more than 20 generations. The father is a Thuringian pet rabbit weighing between 1.5 and 2 kg. Artificial insemination was utilized instead of natural mating due to the significant size difference and to prevent complications. The collected semen was used for the artificial insemination of the Hycole XXL female. The trial took place at a small-scale commercial rabbit farm that produces meat. All animals used in this study were sourced from this commercial rabbit farm. The animals were kept under standard livestock production conditions. All animal procedures were conducted in compliance with ethical guidelines and approved protocols. Prior to the collection of the tissue, animals were euthanized using mechanical stunning and were rendered unconscious, ensuring no pain was experienced during decapitation, complying with standard procedures used in commercial rabbit meat production and compliant with Hungarian animal welfare regulations. No additional chemical anesthetics were administered.

The study involves RNA-seq and Whole-Genome Sequencing (WGS) to investigate genetic and transcriptomic variations, as well as Differentially Expressed Genes (DEG). Four biological replicates were gathered from each individual: three from the back and one from the thigh. Samples for WGS were also collected from the same individuals.

### 2.2. Library Preparation and Sequencing

High-throughput mRNA sequencing analysis was performed on an Illumina sequencing platform to obtain global transcriptome data. The total RNA sample quality was checked by an Agilent BioAnalyzer using a Eukaryotic Total RNA Nano Kit according to the manufacturer’s protocol (Agilent Technologies, Santa Clara, CA, USA). Samples with an RNA integrity number (RIN) > 7 were used for the library preparation process. RNA-seq libraries were prepared from total RNA using an Ultra II RNA Sample Prep Kit (New England BioLabs, Ipswich, MA, USA) according to the manufacturer’s protocol. Briefly, poly-A RNAs were captured by oligo-dT-conjugated magnetic beads, after which the mRNAs were eluted and fragmented at 94 °C. First-strand cDNA was generated by random priming reverse transcription, and double-stranded cDNA was generated by second-strand synthesis. After end repair, A-tailing, and adapter ligation steps, adapter-ligated fragments were amplified via enrichment PCR, and sequencing libraries were generated. Sequencing was performed at the University of Debrecen on an Illumina NextSeq 500 instrument using 150 bp paired-end sequencing (Illumina, San Diego, CA, USA).

#### 2.2.1. Whole-Genome Sequencing

Genome sequencing and library preparation from the *Oryctolagus cuniculus* family samples were performed by Novogene following a standard whole-genome sequencing protocol (paired-end, 150 bp read length) on an Illumina NovaSeq 6000 system (Illumina, San Diego, CA, USA) to an average depth of 35.4 (±6.37).

#### 2.2.2. Quality Control (QC)

A quality control check of the raw fastq files was performed using fastQC v0.11.8 [17]. For the duplication rate check in RNA-seq, the fastp tool was used, as fastQC is more oriented toward single reads and WGS [18].

### 2.3. Generation of an Annotation File for OryCun3.0

OryCun3.0 was chosen as the reference genome, and an annotation file (GTF) was generated using liftoff v1.6.3 software [19] and based on the OryCun2.0 genome as a reference along with its annotation (Ensembl, GTF109, Hinxton, UK), since the OryCun3.0 reference did not have an official annotation. The output is a gff file, which was converted into a gtf file using gffread v0.12.4 [20].

### 2.4. Read Mapping

For ASE detection, we needed to determine the locations from which each read was generated within the reference genome. Therefore, the reads were aligned to the OryCun3.0 genome. We obtained 150 bp paired-end reads from muscle tissue and aligned the reads to the genome using STAR 2.7.1a [21]. First, we generated an index for the genome using the arguments (--sjdbGTFfil and --sjdbOverhang 50) and then performed the alignment using the arguments (–quantMode GeneCounts). With this option, STAR counts the number of reads for each gene while mapping. Both ends of the paired-end reads were checked for overlaps. This option requires an annotation file (GTF) during genome index generation or mapping.

The aligner used for WGS mapping was bwa-mem2 v2.2.1 [22]. SAMtools v1.12 was used to sort and convert the reads to bam files [23].

### 2.5. Read Counts for DEG Analysis

To allow comparison of gene expression across the members of the rabbit family, gene-level counts were analyzed using FeatureCounts v2.0.1 [24], which is suitable and efficient for the general purpose of obtaining sequence reads assigned to the genomic features from bam files, with the optional paired end argument selected (-p).

On the basis of the read counts, DEG analysis between the parents (three mother replicates vs. four father replicates) was performed using the iDEP 2.0 [25] integrated platform with the default parameters and mother vs. father for the DEG section, Genes with fewer than 10 total reads in any three samples were filtered out. PCA was produced using the transformed data from EdgeR v4.0 [26] within the same platform. Similarly, a heatmap for the specified gene set (focused on DEGs) in the parents was produced using DESeq2 v 1.42.1. This set included 773 genes (410 upregulated and 363 downregulated). Gene Ontology Molecular Function (GOMF) analysis was also conducted in the DEG2 section using DESeq2 with default parameters (FDR cutoff 0.1, and a minimum of 1 log2 fold change). A volcano plot was created using the Enhanced Volcano Plot v1.20.0 [27] R package 4.3.2.

### 2.6. Variant Identification

Variants were called from both RNA-seq and WGS data. The Genome Analysis Toolkit (GATK) [28] best practice pipeline for Variant Discovery in High-Throughput Sequencing Data (gatk-package-4.4.0.0-local.jar) was used, although the pipeline slightly differed between RNA-seq and WGS.

Variant calling from the RNA-seq reads was performed on the merged bam files from each individual (four replicates). After STAR mapping, reads with more than two bp soft-clipped at either end were filtered out, and then the best practices GATK [29,30] (MarkDuplicates, Split’N’Trim, base recalibration and applying steps) were performed. Afterward, HaplotypeCaller was used to generate gVCF using the quality threshold of Q 20.0, followed by combining gVCFs and genotyping the final VCF file that contains the variants for the entire family. We also filtered out any variants that had no call (./.) in any individual.

Variant calling from the WGS data began with the mapped reads, followed by MarkDuplicates, BaseRecalibrator, ApplyBQSR, HaplotypeCaller (-ERC GVCF) with default arguments, CombineGVCFs, and GenotypeGVCFs. After these steps, variants were selected from the combined VCF by type (SNP, INDEL, MIXED) using SelectVariants, followed by hard filtering of the variants by type. For SNPs, the following filters were used: “QD < 2.0”, “QD2”, “QUAL < 30.0”, “QUAL30”, “SOR > 3.0”, “SOR3”, “FS > 60.0”, “FS60”, “MQ < 40.0”, “MQ40”, “MQRankSum < −12.5”, “MQRankSum-12.5”, “ReadPosRankSum < −8.0”, and “ReadPosRankSum-8”.

For the INDELs and MIXED variants, the following filters were used (“QD < 2.0”, “QD2”, “QUAL < 30.0”, “QUAL30”, “FS > 200.0”, “FS200”, “ReadPosRankSum < −20.0”, “ReadPosRankSum-20”). Finally, the filtered VCFs were merged again (MergeVcfs), zipped and indexed.

### 2.7. ASE Analysis in the Entire Family

The read count matrix obtained from FeatureCounts for all biological replicates (after removing mother_2, Figure S1) was used as input for read count normalization using DESeq2 v1.42.0 in R [31]. DESeq2 employs methods to test for differential expression using negative binomial generalized linear models. Additionally, the dispersion estimation and logarithmic fold changes incorporate data-driven prior distributions. DESeq2 was further used to estimate contrasts, where each contrast is a linear combination of the estimation of log2 fold changes. Contrasts were created between each individual and the father: [mother vs. father], [offspring_1 vs. father], [offspring_2 vs. father], [offspring_3 vs. father], [offspring_4 vs. father], [offspring_5 vs. father], [offspring_6 vs. father], [offspring_7 vs. father], [offspring_8 vs. father]. The resulting log2fold changes and padj values were then merged for each individual in one large dataset. The final dataset contains log2 fold changes and padj values relative to the values in the father, allowing us to examine the entire family together in one run in the downstream analyses. From this point onward, we extracted the interesting cases in which at least one individual exhibited a [|Log2FC| > 1] and a [padj value < 0.05] and proceeded with the analysis for ASE.

### 2.8. Pinpointing ASE Cases (Genes)

The last dataset (selected interesting cases) was used to identify the genes demonstrating the best and most reliable ASE. In our cases, we refer to high expression with (H), low expression (L), and moderate expression (M), and the notation is given as (Mother_Father). All Log2FC values are calculated relative to the father as the baseline. To classify genes based on their allelic expression ratios, we modeled the distribution of allele ratios using a normal distribution centered at 0 (mean), reflecting the expectation of equal biallelic expression. We defined thresholds for low, medium, and high imbalance by selecting intervals around the mean. We used the 1st and 3rd quartiles of the allelic ratio distribution as thresholds for “low” and “high” imbalance. This data-driven approach provides a consistent framework for classification while also capturing biologically relevant variation (e.g., ±0.4 for medium expression to capture cases around the mean). See Supplementary Materials, Figure S7 for details. The purpose of this stage was to define the ASE cases based on the following criteria:

H_L: Log2FC > 1 in the mother; offspring’s Log2FC spans [−0.2, mother + 0.2] to include all cases of moderate expression and dominant expression when the expression in all the offspring is close to the mother or the father.

L_H: mother’s Log2FC < −1; and 0.2 ≥ offspring ≤ mother −0.2.

H_M/M_L: mother’s Log2FC ≥ 0.8; offspring in two groups (Group 1, Log2FC ≥ 0.8; Group 2, Log2FC ⊆ [−0.4, 0.4]), and at least one of the offspring should have a Log2FC > 1. The idea is that the expression in Group 1 should be similar to that in the mother (H), and that in Group 2 should be similar to that in the father (M). None of the group sets can be empty.

L_M/M_H: mother’s Log2FC ≤ −0.8; Group 1 Log2FC ≤ −0.8; Group 2 Log2FC ⊆ [−0.4, 0.4], at least one offspring is ≤−1.

M_M: mother’s Log2FC ⊆ [−0.4, 0.4]; offspring can be H, M, or L, and at least two groups should not be empty. At least one offspring’s |log2FC| ≥ 1.

Filtering Genes from the Last Dataset According to Exon Coverage Consistency:

The Pysam package (https://github.com/pysam-developers/pysam (accessed on 1 January 2025)) was used alongside a custom Python v.3.8.12 script (https://github.com/Maher199/ASE-in-a-family (accessed on 15 June 2025)) to retain only those genes with at least one exon that has coverage of more than 5 reads at each base pair in that exon, ensuring consistent coverage throughout the exon. 

### 2.9. Identification of Relevant Variants in the Whole Genome and RNA Sequencing

According to our hypothesis, phenotype differences across the family can be attributed to variants in the TFBS (cis-regulatory regions). Therefore, we searched the gene body and the 10 kb surrounding regions for the variants that followed the same pattern as the phenotype using custom Python scripts. We applied separate criteria for each case. Variants in the gene bodies and the 10 kb flanking regions should be as follows:

H_L & L_H: The parents should be homozygous but different (REF or ALT), and all the offspring should be heterozygous.

H_M & L_M: One parent is heterozygous, and the other is homozygous. The offspring should fall into both categories, following the same pattern.

M_M: Both parents should be heterozygous, and the offspring can be in any category concerning the phenotype pattern defined based on the Log2FC.

The same criteria were applied to extract interesting exonic variants via RNA-seq. All previous information about each case for every gene was combined and organized into a dataset that reflects each case.

### 2.10. Identifying Potential Causative Transcription Factor Binding Sites (TFBSs)

The gene of interest and corresponding potential TFBS variants were defined and matched with the consensus TFBS sets from the ChIPSummitDB [32]. These TFBSs were transformed to Orycun3.0 genome coordinates using the following pipeline: The human and rabbit fasta files were first converted to a 2bit format. Using this compact genome representation, each human chromosome was aligned to the rabbit genome using blat. The following parameters were used: -tileSize = 12 -fastMap -minIdentity = 98 -noHead -minScore = 100. The psl format file results were converted to chain files with the axtChain command. We set the linearGap parameter to medium according to UCSC best practice. After that, chainMergeSort and chainSplit commands were used. For chainSplit, set -lump = 50 was set. Afterward, all the chain files were concatenated using the standard ‘cat’ command in Linux and sorted with chainSort. The next step was the creation of chromInfo files from the 2bit files using the twoBitInfo command. Using the chromInfo files and the sorted chain files, a net file was created with the chainNet command. The final step was selecting the correct alignable regions from the net file. The netChainSubset command was utilized for this purpose, and the final liftOver file was used to map human chromosome positions to the rabbit genome. The exact command lines that were used are the following:

faToTwoBit *.fasta oc3.2bit

cd ../../genomes/hg19

../../bin/faToTwoBit *.fa hg19.2bit

./align.sh # use 20 processors

./chain.sh # use 20 processors

../bin/chainMergeSort chains/*.chain | ../bin/chainSplit chainMerge stdin -lump=50

cat chainMerge/*.chain >all.chain

../bin/chainSort all.chain all.sorted.chain

../bin/twoBitInfo hg19/hg19.2bit hg19.chromInfo

../bin/twoBitInfo oc3/oc3.2bit oc3.chromInfo

../bin/chainNet all.sorted.chain hg19.chromInfo oc3.chromInfo net/all.net /dev/null

../bin/netChainSubset net/all.net all.sorted.chain hg19tooc3.liftOver

liftOver humantfbs.bed hg19tooc3.liftOver oc3tfbs.bed unmatched.bed

### 2.11. Haplotype Phasing

We developed a new Python script (https://github.com/Maher199/ASE-in-a-family (accessed on 15 June 2025)) to determine the parental haplotype composition of the given gene regions of each offspring. The script uses stretches of heterozygous sites in one parent, where the other parent is homozygous. The complete description of this haplotype resolution algorithm is explained in (Supplementary Materials, Figure S6).

## 3. Results

To identify and characterize genes that exhibit allele-specific expression (ASE), we designed a combined RNA-seq and WGS experiment. We sequenced a rabbit family consisting of the parents (the mother from Hycole, a meat-producing breed, and the father from a Thuringian breed) and their eight offspring. For each individual, we collected four biological replicates for RNA-seq: three samples from the back and one sample from the thigh. For sequencing, we used Illumina 2 × 150 bp paired-end technology. The number of reads obtained from the 40 samples ranged from 16–28 million. On average, 87% were aligned with the OryCun 3.0 reference genome (See tables in Supplementary Materials, Table S1 for quality control assessments). After quality checking, we noticed that one of the four samples from the mother rabbit generally showed higher expression values. Therefore, we excluded this sample from the mother–father gene expression comparison (Supplementary Materials, Figure S1).

### 3.1. Differences in Gene Expression Between the Two Parents

**Figure 1:**
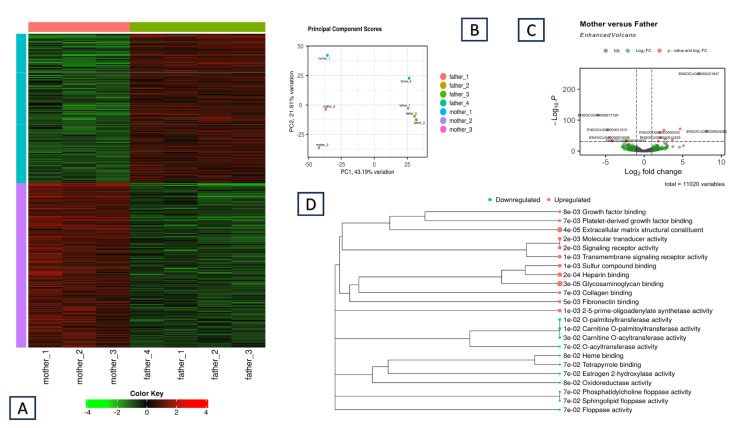
Mother vs. father DEG analysis: (**A**) Heatmap of the expression profile of the gene set of DEGs. Each row is a gene, each column represents a sample, and the sample color is grouped by individual (parents). Red indicates upregulated genes, whereas green indicates downregulated genes. (**B**) Principal component analysis of the three mothers and the four parental expression profiles on the basis of EdgeR analysis in the iDEP. (**C**) Volcano plot. Differential gene expression between the parents, derived from DESeq2. The genes with the most significant differences in expression between the parents (genes in red exceed the –log10 P threshold) and other DEGs (genes in green exceed the Log2FC threshold) are represented. (**D**) GO Molecular Functions (GOMF) enrichment for the upregulated (red) and downregulated (blue) genes in the mother (FDR cutoff 0.1, minimum log fold change of 1).

Our primary aim was to detect and explore ASE in rabbit muscle tissue. For this purpose, we chose two breeds that were genetically divergent from each other [33]. Therefore, the father was a Thuringian pet animal. In contrast, the mother was from the meat-producing Hycole breed, which has undergone intense selection to improve the quantity and quality of the meat. Considering this, we expected a significant difference in the expression level of the muscle-based genes between the two parents. To test this hypothesis, we first generated a gene count matrix between the mother and the father. Next, we conducted a full differential gene expression (DE) analysis on the iDEP2.0 platform using the DESeq2 method [25]. The results are summarized in Figure 1. Overall, we identified 773 differentially expressed genes (DEGs) between the parents, among which 410 genes were upregulated and 363 were downregulated in the mother (Figure 1A). Figure 1C highlights the most significantly differentially expressed genes in an enhanced volcano plot. Among them, the ENSOCUG00000021647 gene had the highest significance. Interestingly, this is a new gene inside an intron of the ENSOCUG00000002686 gene. The complete list of up- and downregulated genes is included along with the GO enrichment analysis (Supplementary Materials, Table S2).

We used the same iDEP 2.0 platform to conduct the GO enrichment analysis (Figure 1D) on these two gene lists. The Gene Ontology Molecular Functions (GOMF) results indicate positive upregulation of several pathways that might be correlated with the phenotypic differences between the parents. These pathways include a growth factor binding pathway and platelet-derived growth factor binding, which could be directly related to muscle growth and meat production through the stimulation of cell and tissue growth and proliferation. The results also included pathways such as extracellular matrix components, skeletal system development, glycosaminoglycan binding, collagen binding, and fibronectin binding. Among the downregulated genes, the most significant hits were related to carnitine metabolism.

### 3.2. Detecting Allele-Specific Expression

We can safely hypothesize that heterozygous sites are needed in regulatory regions to initiate ASE. In the conventional approach, we need heterozygous sites in the transcripts to detect and count ASE. We designed a new experimental setup that is suitable for detecting allele-specific expression without requiring variants in the transcripts utilizing a combination of RNA-seq and WGS data for the pipeline diagram [see Supplementary Materials: Figure S3]. The experimental setup consists of two parents and eight offspring. We hypothesized that we do not necessarily need the heterozygous sites in the offspring transcripts in this family-based model system. However, having such sites would be an advantage in proving the ASE. Therefore, we chose two parents with as many genetic differences as possible (Supplementary Materials, Table S3). The mother Hycole animal had 9.5 million heterozygous (HET) and 18.6 homozygous for alternative allele (HOM_ALT) variants, whereas the Thuringian father animal had 10.4 million (HET) and 18.6 HOM_ALT variants. More importantly, there are 3.3 million sites where one of the parents is HOM ALT and the other is HOM REF. Similarly, there are approximately 13 million sites where one of the parents is HET while the other is HOM. Finally, approximately 2.9 million sites where both parents are heterozygous (HET) were identified, as shown in Table 1.

Assuming the simplest case, where one allele is expressed at a high level and another allele is expressed at a lower level, we can expect that the heterozygous individuals will show intermediate expression. Here, we label high expression with the letter ‘H’, low expression with the letter ‘L’, and moderate expression with the letter ‘M’ (interMediate between the high and low levels). In this case, there are seven possible combinations of parent expression levels for the different genes: H_L, L_H, H_M, M_L, L_M, M_H, M_L, and M_M. In every case, the first letter represents the mother’s allele. In the cases of H_L, H_M, and M_L, we can observe from the parents’ transcripts that the mother has a higher level of expression, whereas we find the opposite in the cases of L_H, L_M, and M_H. In cases with the M phenotype, we hypothesize a heterozygous genotype in a regulatory region. That is, when we compare the expression levels of the parents, we can see only three outcomes: first, the mother has a higher level of expression; second, the father has a higher level of expression; and third (commonly), the expression levels in the two parents are the same.

**Figure 2:**
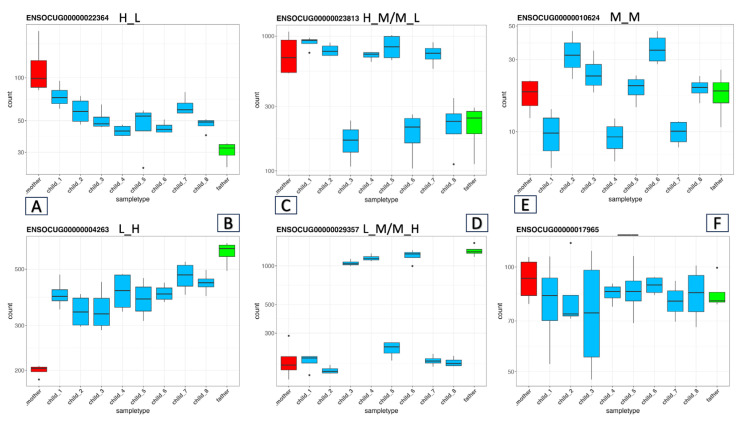
Inheritance Patterns of Gene Expression Across the Family. Examples of the possible gene expression combinations in our family model. The X-axis lists the family members (mother, offspring, and father), and the Y-axis shows the normalized RNA-seq read counts by DESeq2. (**A**,**B**) (H_L) and (L_H) cases, respectively, where the parents have either high (H) or low (L) expression levels, and all the offspring have moderate (M) expression levels, indicating intermediate inheritance. (**C**,**D**): (H_M/M_L) and (L_M/M_H) cases where the offspring are split into two groups, each matching one parent’s gene expression level. The (**E**) (M_M) case demonstrates the otherwise hidden intermediate inheritance where both parents exhibit moderate gene expression, whereas the offspring have H, L, and M expression levels. (**F**) No ASE, that is, genes with no significant differences among any individuals in the family.

The main novel aspect of our family-based approach is that we can evaluate the expression levels of the genes across the whole family. Therefore, if at least one of the offspring has the same expression level as any of the parents, we can safely hypothesize that one or both parents have both the H and L alleles in the regulatory region, which is responsible for the ASE. In other words, if one or both parents are heterozygous at a locus that is responsible for the ASE, that trait will segregate in the offspring. Given that there are eight offspring, the chance that they would inherit the same parental alleles is very small. Thus, by simply determining the expression level of a gene in the two parents and their offspring, we can assign the possible cases of ASE into five categories (Figure 2). According to the logic mentioned above, similar to the parents, we determined the gene expression levels of all eight offspring. We then compared the levels of expression to that in the father as the baseline in terms of Log2FC. By utilizing all this expression information and considering Mendelian laws, we can determine the possible ASE categories by applying the following criteria:

(1) The two parents have significantly different expression levels: |Log2FC| > 1 and *p*-adj value < 0.05. If all the offspring show expression levels intermediate (M) between those of the parents, then we can assign these genes to the H_L or L_H categories (Figure 2A,B) depending on which parent’s expression was higher (the first letter refers to the mother).
(2) The parents have different expression levels; the individual offspring’s expression levels are distributed into two categories, which align with the parents’ two expression levels; and at least one individual has a |Log2FC| > 1 and a *p*-adj value < 0.05. In this case, we can assume that one of the parents is under heterozygous regulation (M). The (H_M, L_M) and (M_H, M_L) cases fall into this category (Figure 2C,D).
(3) There is also a special case where both parents’ expression levels are similar (Log2FC in the range of [−0.4, 0.4]), but in accordance with the Mendelian law of segregation, the expression levels of the offspring fall into three categories (Figure 2E); some will be above (H), some will be below (L), and some will be similar (M) to the parent’s expression level. Although eight offspring are not a sufficient number for detailed statistical analysis, the ratios of the three cases should adhere roughly to Mendel’s law of segregation for the F2 generation (1:2:1). Here, the second category, with a twofold ratio, should correspond to the offspring whose expression level is similar to that of the parents.
(4) In many cases, the parents’ expression levels will be similar and the expression levels in the offspring will not segregate. In these cases, we can assume that no ASE exists (Figure 2F).

According to this logic, we set up these criteria in a script that takes the expression values of the two parents and the eight offspring at each gene and predicts the existence and possible type of ASE. After applying very strict filtering, this analysis predicted 97 H_L, 104 L_H, 469 M_M, 110 H_M or M_L, and 133 L_M or M_H genes (Table 2). The expression plots are in the repository https://doi.org/10.5281/zenodo.14794348. The normalized read counts and Log2FC values (with the father as baseline) and the predicted ASE types for each gene can be found in Supplementary Materials, Table S4. Looking at the numbers, it is remarkable how high the number of M_M cases is. Interestingly, only eleven genes have at least one offspring in each of the three categories (H, M, and L). In these cases, we hypothesize that the parents are both heterozygous for at least one regulatory site. Therefore, this variant will segregate in the offspring. Ideally, among the eight offspring, we should see two L, four M, and two H cases, but of course, the random inheritance of the parental alleles can produce other ratios (such as 3:5:0), making detailed statistical analysis challenging for confirming strict Mendelian proportions.

### 3.3. Validation of the Predicted ASEs

**Figure 3:**
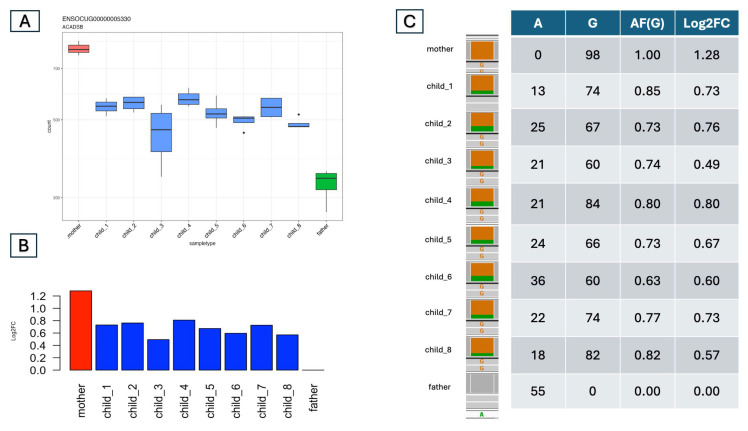
The allele-based read-counting ratios were in line with the expression-based fold changes. (**A**) Normalized RNA-seq read counts across the whole family. The x-axis represents individual family members, and the y-axis represents the normalized read counts. The mother is H, the father is L, and all the offspring are M. (**B**) Log2 fold change (Log2FC) values. The expression in the father is set to zero. The mother is H (>1 Log2FC). (**C**) A table comparing the allele ratios to the fold changes. An A/G heterozygous variant was identified in the exon of the ACADSB gene: the mother is HOM_ALT (G/G), whereas the father is HOM_REF (A/A). All of the offspring are heterozygous (G/A). The table presents the allele read counts for this variant. The read count ratio (allele frequency) is calculated and compared with the Log2FC values in the final column.

The above-described analysis is based on only the expression levels of each gene. In the traditional approach involving crossing two breeds and then performing RNA-seq of the offspring, ASE can be detected only if at least one heterozygous site exists in a transcript. We can also count the reads bearing one or the other allele in these cases. The difference is that we can perform allele counting not only for one individual but also for all eight offspring and their parents. Then, we can compare these results with the differences in expression. After identifying variants via RNA-seq, we examined the allele ratio at each heterozygous variant in the exonic regions of the ASE genes identified with our method to validate our bulk expression-based predictions (see the complete set of comparisons in Supplementary Materials, Table S4). As an example, we chose the simple case of the acyl-CoA dehydrogenase short/branched chain (ACADSB) gene from the predicted H_L cases (Figure 3). Here, the expression data based on normalized read counts (Figure 3A) and father-based log2fold changes (Figure 3B) clearly show that, indeed, all eight offspring have expression levels between those of the mother and the father. Although this result clearly indicates ASE, we also examined the allele ratios in the offspring. Figure 3C shows the results of this analysis. There is a heterozygous (A/G) variant in the transcript of this gene. The mother is homozygous for the alternative allele (HOM_ALT) (GG), the father is homozygous for the reference allele (HOM_REF) (AA), and the offspring are all heterozygous (HET) (AG). The allele frequency of G in the offspring is between 0.63 and 0.85, which agrees well with the measured Log2FC (Figure 3C). Therefore, we used the same approach to check all the predicted ASEs for transcript variants.

Table 2 summarizes the case numbers along with the variants discovered. Surprisingly, we found variants in only a small fraction of the predicted ASE genes. For example, out of 469 predicted M_M cases, only one gene contained a heterozygous variant in both parents in the RNA-seq data. On the other hand, in most cases, there was more than one variant. As in the above-described genes, the allele ratios were consistent with the changes in the expression values.

In general, we hypothesize that a variant in a cis-regulatory region (AB) can produce three gene expression phenotypes. The AA genotype is responsible for the H phenotype, the AB genotype is responsible for the M phenotype, and the BB genotype is responsible for the L phenotype. This means that if we see, for example, an H_M ASE, then the parental genotypes must be AAxAB, and the offspring should have either the AA or the AB genotype. In the same example, if the father has other heterozygous variants in the given wider region, then those variants must show the same inheritance pattern among the offspring (assuming that there was no recombination in the given region). Therefore, finding variants that show the same pattern as the predicted ASE type can support our original expression-based ASE predictions.

To carry out this analysis, we sequenced the whole genomes of all 10 animals with ~30× coverage (Supplementary Materials, Table S1). After mapping and variant calling, we examined the variants in the ASE-predicted genes. We considered three regions: the transcripts (essentially what we observed in the RNA-seq), the introns, and the 10 kb regions surrounding each gene. Supplementary Materials, Table S6 shows the number of variants that support the expression-based ASE predictions at these three regions at each ASE-predicted gene. The results are summarized in Table 2. The predicted ASEs were confirmed in 56.7% and 62.5% of the H_L and L_H cases, respectively. In these cases, there was at least one variant in these regions for which both parents were homozygous and all offspring were heterozygous. This finding matches perfectly with the observed M expression phenotype in the offspring. In the H_M or M_L and L_M or M_H categories, we found confirming variant patterns at only 29.2 and 33% of the 119 and 133 ASE-predicted genes, respectively. Surprisingly, this ratio was much lower in the 469 predicted M_M ASE cases, where only 7.3% of the genes presented at least one confirming variant. In this analysis, we found that different combinations of variants can exist in many genes, especially if they have long introns and/or many exons. Supplementary Materials: Figure S3: shows the example of the BDH2 gene, which is a lipid deposition-related gene, as reported by Wang et al. [34]. On the basis of the gene expression data of the parents and offspring (panel A), we initially assigned this BDH2 gene to the L_M or M_H category (Supplementary Materials, Table S4). According to our hypothesis, this implies that one of the parents must be heterozygous at a site where the other parent is homozygous. Surprisingly, when we inspected the overlapping variants, we found examples for each type of combination, which involved at least one heterozygous variant in the parents (Supplementary Materials: Figure S3D). In this panel, the first column shows an IGV screenshot of a variant where both parents are heterozygous, and the alleles segregate in F2 at a 1:6:1 ratio. This variant cannot explain the observed expression pattern because the expression levels are different in the parents. The second column of Panel D shows that the mother is HET (A/G), whereas the father is HOM (G/G). From this, we can assign the gene to the M_H category. However, when we compared the variant pattern of the offspring to the expression pattern, we found that they did not match. Finally, the last column of Panel D shows an exact match to the observed expression pattern and thus confirms our ASE prediction. Here, the mother is HOM_REF (AA), and the father is HET (A/G). The eight offspring are HOM, HOM, HET, HET, HOM, HET, HOM, and HOM, which align perfectly with the observed M, M, H, H, M, H, M, and M expression patterns, respectively. To further confirm the expression-based prediction of the ASE category, we developed a script (https://github.com/Maher199/ASE-in-a-family (accessed on 15 June 2025) and Supplementary Materials, Figure S6) that groups offspring according to the parental chromosome they inherited. The script utilizes sites where one parent is heterozygous and the other is homozygous. Assuming that the gene is under regulation by a cis-acting element, the outcome of this haplotype phasing must align with the predicted ASE pattern. This is illustrated in Supplementary Materials: Figure S3. Here, offspring 1, 2, 5, 7, and 8 inherited the red (A) allele from both parents. Therefore, they are homozygous for the low allele, resulting in a low expression level. In contrast, the other three offspring inherited the green (B) allele from the heterozygous (M) father, making them heterozygous for the cis-acting element, which led to an M expression level.

This analysis also allows us to choose the actual ASE in the H_M or M_L and L_M or M_H cases. For example, for this BDH2 gene, we cannot say whether it is an L_M or M_H ASE on the basis of expression analysis alone. However, after examining the variant patterns and the phasing results, we found only one match, where the mother was homozygous. This confirmed unanimously that this is an L_M ASE because the M parent must be heterozygous. Accordingly, the offspring are L, L, M, M, L, M, L, and L in that order.

M_M cases are unique in that they cannot be observed without inspecting the expression levels of the offspring, and at least one offspring should be homozygous. Supplementary Materials: Figure S4 provides an example of a new gene that exhibits all possible expression phenotypes among the offspring (L, H, and M), and variants and haplotype phasing support the expression pattern. Continued analysis of M_M cases led us to find some interesting cases where all but one offspring exhibited moderate expression (M) and the exception exhibited either high (H) or low (L) expression. In the example gene (Supplementary Materials: Figure S5), the B allele, which is responsible for low (L) expression, matched with the father’s B allele only in the first offspring. We could only impute the haplotype of this gene after extending the phasing region beyond the gene borders because there were no heterozygous variants within the gene body in the mother.

### 3.4. Looking for Regulatory Variants

The availability of whole-genome sequencing data enabled us to test the hypothesis that at least one heterozygous variant must be present in a regulatory region for allele-specific expression. To identify such regulatory SNPs (rSNPs), we first needed to predict the transcription factor binding sites (TFBSs) in the rabbit genome. Owing to the lack of comprehensive ChIP-seq-based TFBS genomic mapping, we utilized our human ChIPSummitDB database [32]. First, we aligned the whole human hg19 reference genome to the rabbit OryCun3.0 reference genome sequence. Next, using the chain files and our human ChIP-seq-based consensus transcription factor binding site collection, we determined those TFBSs that are conserved between the human and rabbit genomes. Finally, we examined the ASE-predicted genes and their 10 kb surrounding regions for variants in the predicted TFBSs, which exhibited the same genotype pattern across the family as that observed in the expression values. The results of this analysis are summarized in Table 3 and detailed in Supplementary Materials: Tables S7 and S8. We identified 222 conserved TFBSs in all 90 genes, all of which contain a variant with an inheritance pattern identical to that predicted for the given ASE gene.

**Figure 4:**
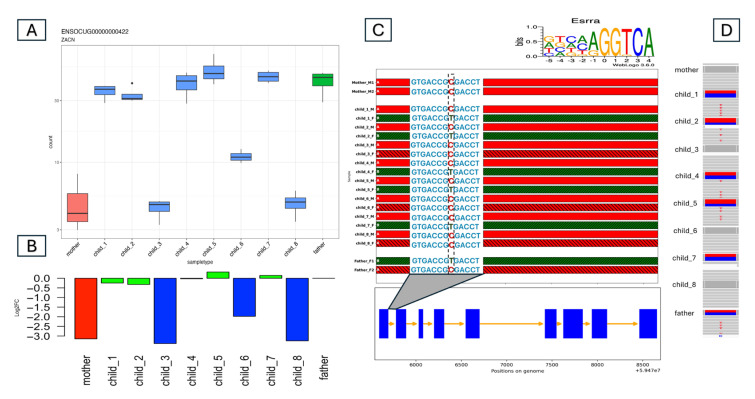
ZACN Gene ASE Analysis Demonstrating an Example of the L_M Expression Pattern Across Family Members. (**A**) Normalized RNA-seq read counts with color-coded members: mother (red), father (green), and offspring (blue). The mother and three offspring (3, 6, and 8) exhibit low (L) expressions, whereas the father and the remaining offspring exhibit moderate (M) expressions. (**B**) Log2 fold change (Log2FC) relative to the father. The low-expressing (L) individuals display a Log2FC of <−0.8, whereas the M-expressing individuals have a Log2FC close to zero, as shown on the y-axis. (**C**) Haplotype phasing of the ZACN gene across the family with haplotype panels colored according to parental imputation. The y-axis lists the family members, and the x-axis shows the expanded gene region position in the genome. A conserved TFBS for Esrra is highlighted in the first intron with the variant C/T in the M-expressing individuals. The consensus sequence for Esrra from ChIPSummitDB is shown above the plot. (**D**) IGV view of the variant in the conserved TFBS. The mother and the L-expressing offspring are homozygous (C/C), whereas the father and the M-expressing offspring are heterozygous (C/T).

Figure 4 shows an example of a possible cis-acting element. We predicted that the ZACN gene has L_M or M_H allele-specific expression on the basis of the gene expression data. Mothers and offspring 3, 6, and 8 presented lower gene expression levels than did fathers and other offspring. In the first intron of this gene, we found a conserved Essra binding site. That is, an Esrra antibody-based human ChIP-seq experiment identified a peak at this position, and the peak region contained an Esrra binding site motif. The rabbit OryCun3.0 reference genome contains a similar motif (AGGTCgcGGTCA, capital letters match the consensus site) in this conserved position. (A/G) GGTCA [35] is the consensus binding site for a nuclear hormone receptor, so it is an incomplete DR0 nuclear hormone receptor dimer binding site. The mother is HOM_REF at this variant, whereas the father is HET. Among the offspring, only offspring 3, 6, and 8 remained homozygous, perfectly matching the ASE pattern. This also means that this is a case of L_M-type ASE. Interestingly, the ALT (A) allele from the father produces an AGGTCAcGGTCA nuclear hormone receptor site, which now contains a perfect first half site. We hypothesize that this variant also thereby results in the increased expression observed in the father and in offspring 1, 2, 4, 5, and 7.

## 4. Discussion

Allele-specific expression (ASE) occurs when the expression level of a given gene differs between the maternal and paternal chromosomes. The existence of ASE implies that the expression of a given gene is under different regulation at the maternal and paternal alleles.

Most key ASE findings have been based on counting overlapping reads on a variant in a transcribed region, usually utilizing RNA sequencing samples from an F1 offspring after crossing two genetically distant parents [36,37]. The limitation of this approach is that it requires at least one heterozygous site in the transcribed (and therefore sequenced) region. However, exonic variants do not exist for all genes. We found that approximately 19% of expressed genes (2440/12,659) did not contain any reliable variants in the RNA-seq reads. The ExAC project genotyped 91,000 human exomes and reported that 99% had a variant frequency of less than 1%, and 50% of variants were singletons [38]. Approximately 30% of genes have only one heterozygous exonic variant within the 1000 Genomes Project database [15]. Moreover, when gene expression is very low, heterozygous variants might be mistakenly called homozygous. The other approach (eQTL finding) involves both RNA and WGS sequencing of many individuals to find variants where the genotype pattern (AA, AB, and BB) is in line with the given expression level [39].

To our knowledge, rabbits have not been used previously for ASE studies. We chose rabbits because of the availability of two genetically divergent breeds with relatively high numbers of heterozygous variants [33]. In this work, we showed that a family-based approach can be used to detect ASE without existing variants in the transcribed regions. Our method also helps predict regulatory variants responsible for controlling gene expression level without resequencing many individuals.

For this analysis, we chose a meat-producing breed (Hycole) for the mother and a Thuringian hobby rabbit as the father for mating. The use of parents from two breeds with relatively distant genetic backgrounds ensured that more variants would be found between the parents’ haplotypes in the offspring. First, we analyzed the differentially expressed genes (DEGs) between the parents. We reported several key genes that might contribute to the phenotypic difference between the meat-producing mother and the pet breed father. Notably, according to the Gene Ontology analysis, muscle-related genes were not directly enriched among the genes whose expression was upregulated in the mother. Among the DEGs identified, for example, the catalase (CAT) gene was downregulated in the father. This enzyme is released in response to oxidative stress and reduction, alongside acyl-CoA oxidase 2 (ACOX2), which is responsible for fatty acid metabolism. Moreover, it downregulates estrogen 2-hydroxylase activity. Rabbit meat has a relatively low fat content. This trait is critical for the quality of the meat. Therefore, understanding the regulation of fatty acid metabolism in the muscle is important for breeding. We also identified several other differentially expressed genes that have been reported in previous studies in meat and muscles. The aldehyde oxidase 1 gene (AOX1) was also upregulated in the mother. Previous studies [40,41] reported that this gene encodes a homodimeric protein that plays a critical role in muscle development in farm animals. Other DEGs included KCTD15, UCPs, SESN1, MGP, PLBD, and BDH2. The complexity of these traits is such that while some genes in the mother work to increase the size of the animal, they also decrease the need to metabolize fat and regulate body temperature. This phenomenon is often observed in wild-type animals and may have developed during breeding.

We developed a novel pipeline combining WGS and RNA-seq in a large animal family to discover and characterize ASE. The pipeline does not necessarily require heterozygous variants in the gene body. Instead, it relies on comparisons of gene expression (phenotype) across the family members. ASE predictions are supported by Mendelian haplotype phasing and by identifying variants that match expression patterns. These variants can be transcribed (and thus detected in the RNA-seq or WGS reads) or in the noncoding intronic and surrounding intergenic regions (detected in WGS reads only).

Without an available rabbit transcription factor binding site (TFBS) database, two general approaches can be used to identify possible cis-eQTLs (TFBSs with regulatory SNPs). The first involves searching for candidate binding sites de novo. This approach may lead to an excessive number of false positive hits. The second, based on the observation that about 2 million human TFBSs are under mammalian constraint [42], examines conserved TFBSs. Therefore, based on our human ChIPSummitDB database consensus binding site catalogs, (https://summit.med.unideb.hu/summitdb/ (accessed on 1 June 2025)) we identified conserved rabbit transcription factor binding sites. We assumed possible cis-regulation if a variant in a conserved TFBS showed the same inheritance pattern as the nearby ASE-predicted gene. These potential TFBSs can be utilized in future studies on meat quantity and quality in farm animals, especially in rabbits.

Assuming that a regulatory variant causes high or low expression levels, we can expect seven different combinations of the three expected gene expression levels (L, M, and H). However, based on the expression data alone, we can distinguish only 5 + 1 different types (Figure 2). In this family-based model, we found examples of all of these categories. Among them, the most straightforward cases are where both parents are homozygous (H_L or L_H). It is also simple when both parents are heterozygous (M_M) because we can analyze this case as an F1 cross and expect that the expression level of the given genes will segregate accordingly in the offspring. Although these cases are understudied, since they exhibit similar phenotypes and genotypes in parents, they can still cause considerable differences in the expression levels in the offspring. The problem in the other two cases, where one parent is homozygous while the other is heterozygous, is that we do not know which parent is M.

Accurately defining cis-regulatory elements that have potential influences on gene expression remains challenging. Moreover, distinguishing the impacts of these elements from those of trans-elements has yet to be accomplished [43]. Our family-based setup offers a simple way of pinpointing potential cis-acting elements without sequencing many individuals. Establishing the expression patterns of the genes within a family provides an easy way to find the matching variants nearby. Knowing the conserved TFBSs allowed us to predict possible cis-acting variants. On average, we found that 21% of the predicted ASE genes were potentially regulated by cis-acting elements. Thus, 79% of expression differences may be due to distant rSNPs or regulatory variants on other chromosomes affecting transcription factors, with some cases involving multifactorial regulation. Our results align with other studies that showed that cis elements in mice regulate 12–24% of genes [44,45]. We found that the H_L and L_H categories showed the highest cis-regulation rates (56% and 62%), while M_M cases had the lowest (7.3% in nearby TFBSs; Tables S7 and S8). The latter may reflect complexity of these cases. Importantly, in these special cases, the given gene has the same expression level in both parents, whereas the offspring may present two or three different expression levels. Aside from the trivial cis-regulated M-M ASE, the potential reasons for this phenomenon can be quite complex and require further investigation.

We are confident that this pipeline could be further improved using long-read sequencing and reciprocal crosses to remove parent-of-origin effects. The methodology explained in our study can serve as a pipeline for genetic studies in families aiming to characterize the ASE phenomenon, a potential source of phenotypic variation in disease and commercial traits. Moreover, the variants in the TFBSs reported in this study can serve as reference points for cis-acting elements that regulate muscle and growth development in rabbits. In summary, our method can provide a comprehensive pipeline for characterizing ASE genes within a large family, aiming to identify ASE genes and potential cis-acting elements. We applied this method to a rabbit family using muscle RNA-seq and WGS. In addition to providing a detailed ASE analysis, we identified potential genes responsible for the increased meat quantity and quality in the Hycole breed.

### Study Limitations

In our experimental setup, we studied a rabbit family with eight offspring. Naturally, this limits a detailed statistical analysis of the allele ratios, especially for the M_M cases. However, even with an expected 1:2:1 segregation ratio, we can safely expect at least one example in each phenotype category. Additionally, for ASE categorization, we do not need to see the exact allele ratios—only to match the inheritance pattern to one of our assumed cases.

In this study, we found that only 21% of the predicted ASE genes were potentially regulated by cis-acting elements identified through variants. This indicates that for a large majority (79%) of the predicted ASE genes, the method was unable to provide enough genetic evidence for cis-regulation within the defined search regions (gene body and 10 kb flanking regions). This applies particularly for the M_M (moderate in both parents) cases, which constitute the largest group of detected ASE genes (469 out of 913 total ASE genes). Only 7.3% of the 469 predicted M_M ASE genes presented at least one variant that matched the expression pattern. In contrast, H_L and L_H cases had much higher confirmation rates (56.7% and 62.5%, respectively). The reason for this high number of genetically unconfirmed cases varies, and it is the main limitation of the study. Obviously, these can be false-positive ASE genes. It is also possible because during the development of the method, we intended to minimize the false negatives by widening the middle range (see Supplementary Materials: Figure S7). However, we are convinced that these cases can represent real scenarios where both parents have a baseline expression level, and we see a full or half segregation in the offspring. This can be caused by a heterozygous variation in a relevant, most likely trans-regulatory region or protein in one or both parents.

## 5. Conclusions

In this study, we introduce a new approach for identifying genes with allele-specific expression (ASE) in either parents or offspring. This study presents a proof-of-concept family-based analysis for detecting allele-specific expression. Our approach is also suitable for predicting the cis elements (i.e., mapping the possible eQTLs) that may be responsible for driving the given ASE. This method has an advantage over other published methods because it does not require the transcripts to contain heterozygous variants or the sequencing of many individuals. The pipeline has great potential for use in animal and plant breeding. Our innovative algorithm for phasing haplotypes within a family can be further developed into a program that accepts variants as input and outputs the haplotypes of the offspring, along with the recombination regions and near-chromosomal level haplotype sequences of the parents. While the method shows promising results in a moderately sized dataset, further validation in additional populations and experimental systems is required to establish its generalizability and robustness. Although our results are mostly convincing, having more offspring could further enhance the accuracy of these types of studies.

## Figures and Tables

**Table 1:** Summary of parent variants: Summary of WGS variant counts in parents considering all possible combinations of homozygosity and heterozygosity between the parents considering the allele in the OryCun3.0 reference genome as A.

Reference	A	A	A	A	A	A	A	A
Mother	AA	BB	BB	AB	AA	AB	BB	AB
Father	BB	AA	BB	AB	AB	AA	AB	BB
Var_Count	1,612,167	1,693,817	13,833,792	2,896,419	4,198,928	3,657,972	2,765,210	2,439,608

**Table 2:** Summary of ASE cases. A summary table of ASE genes, including the numbers and percentages of evident variants matched at each level of the analysis. The table summarizes the number of genes that were found to have variants in each expression category (H_L, L_H, M_M, H_M or M_L, and L_M or M_H) and the number of matched variants identified at the corresponding level of evidence: RNA variants, DNA in the exonic region, DNA in the intronic region and DNA in the surrounding 10K regions upstream and downstream. The last row shows the number and percentage of the sum of the unique genes after accounting for the overlaps.

Cases	H_L			L_H			M_M			H_M or M_L			L_M or M_H		
No. Genes	97			104			469			110			133		
	All	Matched	%	All	Matched	%	All	Matched	%	All	Matched	%	All	Matched	%
RNA	65	27	41.5	72	35	48.6	341	1	0.3	72	3	4.2	94	8	8.5
DNA_EXON	81	34	42	88	45	51.1	374	1	0.3	89	6	6.7	105	14	13.3
DNA_INTRON	84	42	50	97	60	61.9	423	16	3.8	103	14	13.6	116	24	20.7
DNA_Outside	97	49	50.5	103	61	59.2	462	13	2.8	110	17	15.5	132	21	15.9
No. of Unique Genes	97	55	56.7	104	65	62.5	467	25	7.3	110	21	29.2	132	31	33

**Table 3:** TFBS Summary: Summary of potential transcription factor binding site (TFBS) analysis. The table indicates the number of transcription factors (TFs) with binding sites overlapping variants that have the same patterns as the corresponding expression in both the intronic region and the 10 kb surrounding regions. The table also shows the number of potential target genes.

	TFs					
	H_L	L_H	M_M	H_M/M_L	L_M/H_M	Sum
Surrounding_regions	24	43	11	5	14	97
Introns	33	83	0	2	7	125
	Genes					
Surrounding_regions	11	22	1	3	6	43
Introns	11	30	0	1	5	47

## Data Availability

All generated sequencing data have been submitted to NCBI, Sequence Read Archive (SRA) with the following BioProject accession number PRJNA1237607. Sequencing runs can be accessed individually with the SRA accession numbers starting from SRX28036935. The father’s sample was submitted earlier at the PRJNA1118464/SRS21521871 accession number. Source code for complete RNA-seq and WGS pipelines and scripts used for allele-specific expression analysis, besides the count plots for all ASE genes discovered, are available from GitHub at https://github.com/Maher199/ASE-in-a-family (accessed on 15 June 2025) and in archived form at https://doi.org/10.5281/zenodo.14794348.

## Supplementary Materials

The following supporting information can be downloaded at: https://www.mdpi.com/article/10.3390/ani15182766/s1, Table S1: RNA-seq and WGS sample quality control and alignment; Table S2: Lists of up- and downregulated genes and GO enrichment analysis; Table S3: List of heterozygous and homozygous variant numbers in the family in the WGS; Table S4: Lists of predicted ASE genes in their categories along with normalized counts and Log2FC values; Table S5: Comparison (lists) of results from our approach and the conventional methods for the variants found in the transcripts; Table S6: Lists of the numbers of complete variants and matched variants at each ASE gene with respect to their expression category; Table S7: Variants discovered in the TFBS in the gene’s surrounding regions; Table S8: Variants discovered in the TFBS in the gene’s intronic region. Figure S1: DEG comparison of parental samples before mother_2 sample elimination; Figure S2: Pipeline overview; Figure S3: BDH2 gene with allele-specific expression analysis across the family members; Figure S4: Novel gene (ENSOCUG00000010624) expression analysis across family members; Figure S5: SLC2A11 gene ASE analysis in the family model; Figure S6: phase_M.py haplotype phasing methodology; Figure S7: Gene Classification into expression categories.

## Abbreviations

The following abbreviations are used in this manuscript:

ASE	Allele-Specific Expression
DEG	Differentially Expressed Gene
TFBS	Transcription Factor Binding Site
WGS	Whole-Genome Sequencing
