# Supplementary material for: A New Family-Based Approach for Detecting Allele-Specific Expression and for Mapping Possible eQTLs
Source: Animals (Basel). 2025 Sep 22;15(18):2766. doi: 10.3390/ani15182766 (PMC12466419; doi:10.3390/ani15182766)
Supplement: Supplementary file 1 [file animals-15-02766-s001.zip › Figure S1.pdf]

We excluded the sample mother_2 from the DEG analysis between the parents due to its outlier gene expression values. Figure S.1 shows the following: A, Heatmap of the complete set of differentially expressed genes before elimination of mother_2. This clearly shows the outlier expression in mother_2, as they are a brighter red. In addition, the number of upregulated genes was significantly greater than that in the final analysis without the mother_2 sample. B, Correlation matrix showing a linear relationship among the samples. The correlation matrix was constructed for each individual using the cor and corrplot functions in R after a variance stabilizing transformation (VST) was performed in R using the DESeq2 library. This figure illustrates the low correlation of the mother_2 sample compared with the other replicates.

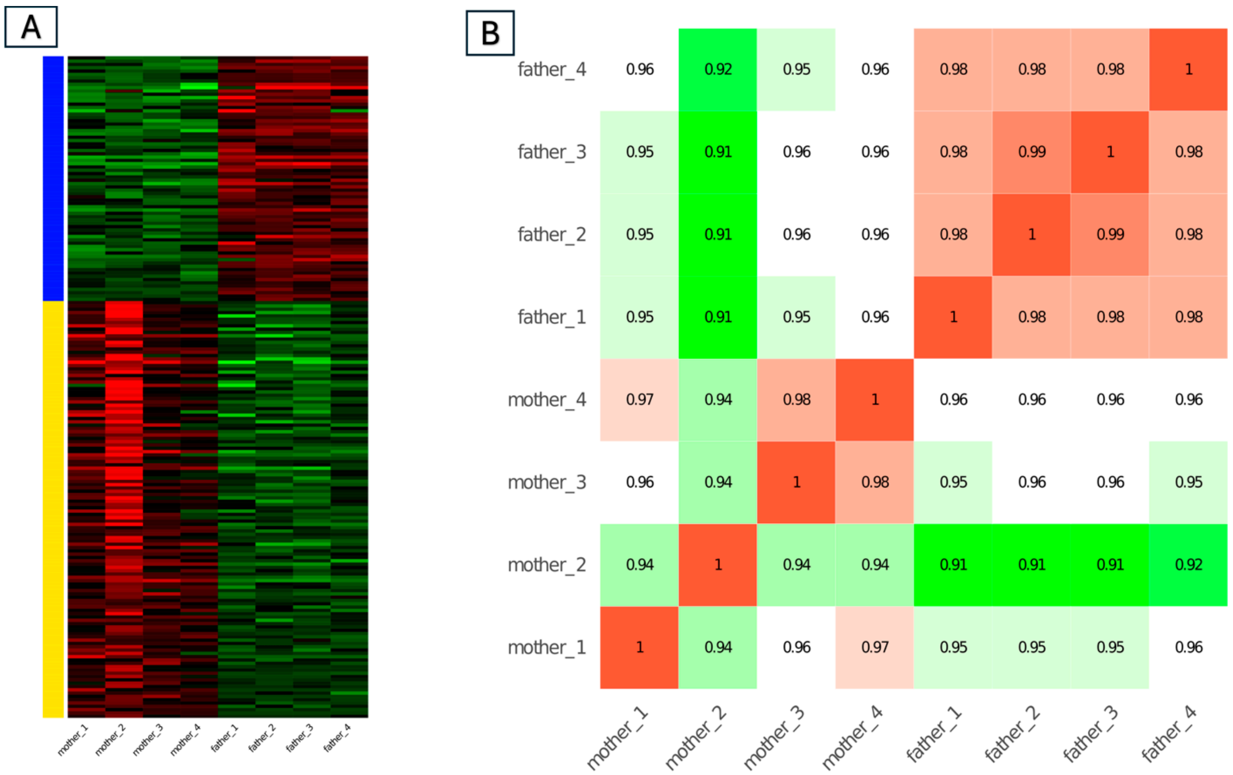

Figure S1: Initial DEG analysis before the removal of the mother_2 sample. A: Heatmap of the DEGs, red indicates upregulated genes and green indicates downregulated genes in the mother vs. father comparison. B: Correlation matrix between the parent samples. Red indicates the highest correlation, and green indicates the lowest correlation.
