# Supplementary material for: A New Family-Based Approach for Detecting Allele-Specific Expression and for Mapping Possible eQTLs
Source: Animals (Basel). 2025 Sep 22;15(18):2766. doi: 10.3390/ani15182766 (PMC12466419; doi:10.3390/ani15182766)
Supplement: Supplementary file 1 [file animals-15-02766-s001.zip › Figure S2_3_4_5.pdf]

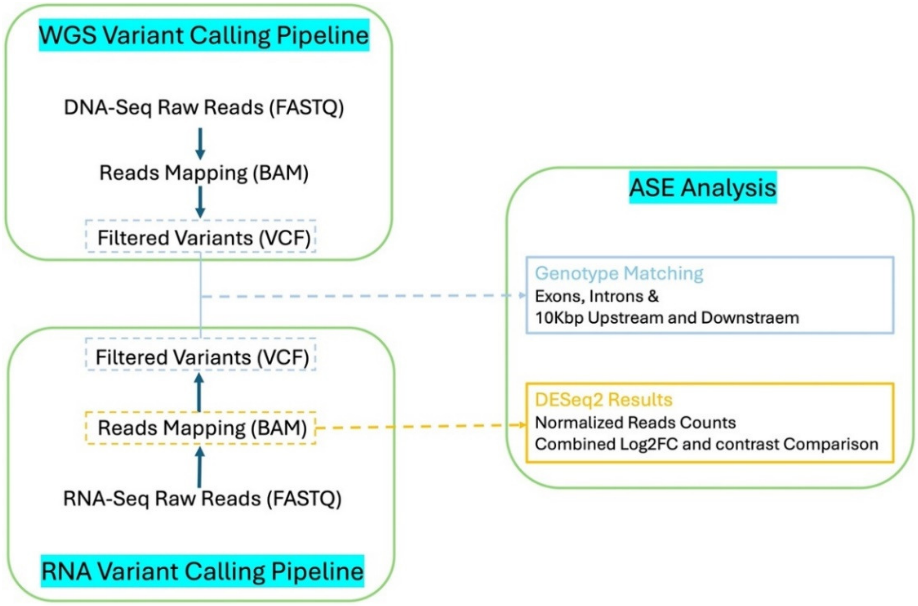

Figure S2: Pipeline overview. Calling variants from both RNA-seq and WGS. WGS in the noncoding region was used to define cis-regulatory elements. RNA-seq was also used to create a read count matrix.

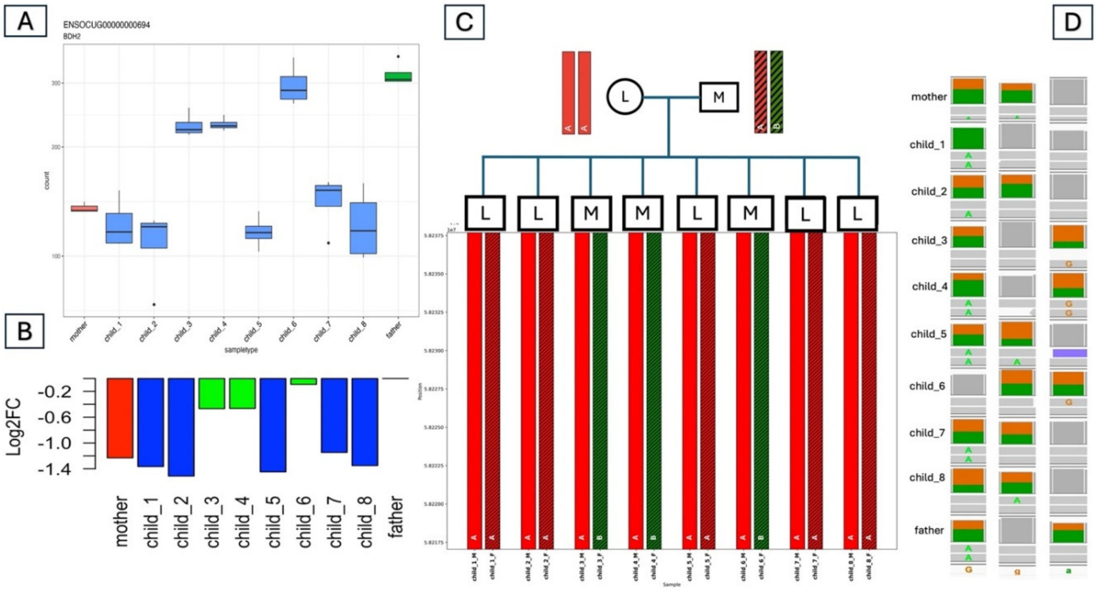

Figure S3: BDH2 gene with allele-specific expression analysis across the family members. A) Normalized read counts for all family members. The mother is shown first, in red; the father is shown last, in green; and the offspring are shown in between. B) Log2FC comparison in the family relative to the father as the 0 baseline. The mother is shown in red, and offspring with <= - 0.8 log2FC are in blue. C) Haplotype phasing in the family; low expression (L) is homozygous (AA), and moderate expression (M) is heterozygous (AB). D) IGV snapshots for three different genotype combinations of variants in the same gene; only the last variant matches the expression pattern of the mother (AA) and father (AG).

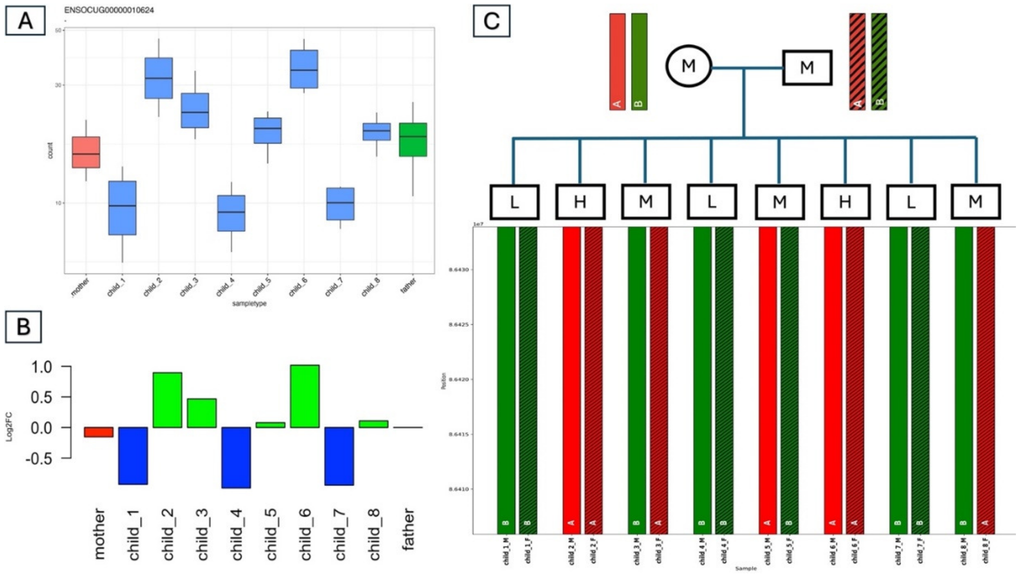

Figure S4: Novel gene (ENSOCUG00000010624) expression analysis across family members. A) Normalized read counts for all family members. The mother is shown first, in red; the father is shown last, in green; and the offspring are shown in between. B) Log2FC comparison in the family relative to the father as the 0 baseline. The mother is shown in red; offspring <= -0.8 are in blue and reflect (L). C) Haplotype phasing at the gene region. Heterozygosity (AB) is in accordance with (M) expression in offspring (3, 5& 8); (AA) reflects (H) expression in offspring (2& 6); and (BB) reflects (L) expression in offspring (1, 4& 7).

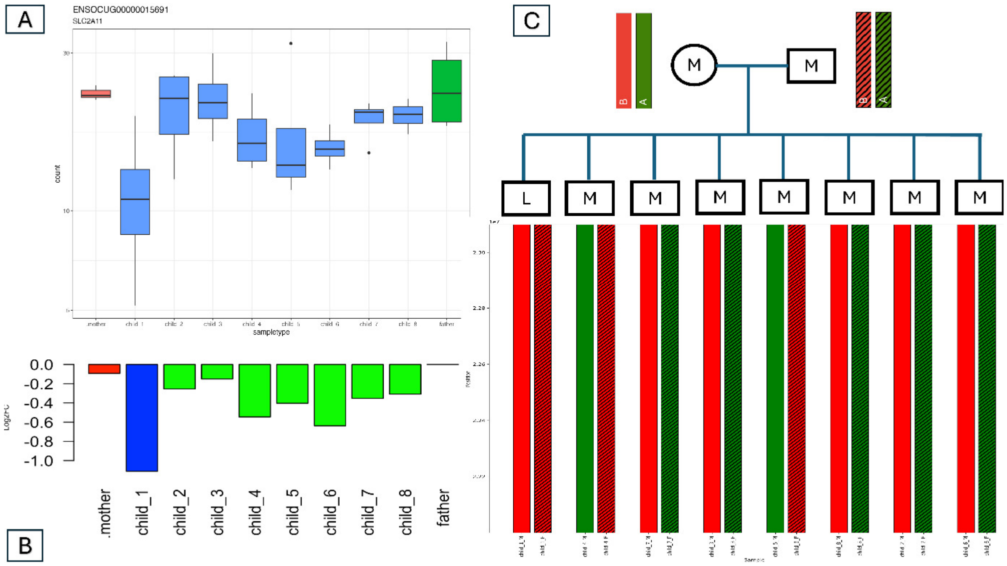

Figure S5: SLC2A11 gene ASE analysis in the family model. A) Normalized read counts for all family members. The mother is shown first, in red; the father is shown last, in green; and the offspring are shown in between. All the offspring have an (M) expression, except for offspring_1 with (L) gene expression. B) Log2FC comparison in the family relative to the father as the 0 baseline. The mother is shown in red, and offspring_1 is shown in blue, with low expression and log2FC values (<= - 0.8). C) Extended haplotype phasing beyond the gene region. Only offspring_1 is homozygous (BB) consistent with the L expression, and heterozygosity in the remaining offspring is consistent with the M expression.
