# Supplementary material for: A New Family-Based Approach for Detecting Allele-Specific Expression and for Mapping Possible eQTLs
Source: Animals (Basel). 2025 Sep 22;15(18):2766. doi: 10.3390/ani15182766 (PMC12466419; doi:10.3390/ani15182766)
Supplement: Supplementary file 1 [file animals-15-02766-s001.zip › Figure S6.pdf]

Haplotype Phasing:

The program phase_M.py phases the parents' haplotype blocks in the offspring and reports the separate parent haplotypes in each offspring. The algorithm depends on blocks of heterozygosity. For the program to work properly and provide accurate phasing information, there must be heterozygous (HET) variants in one parent and homozygous (HOM) variants in the other parent in the given region; if not, the region should be extended to ensure that this condition is met. Additionally, the program outputs a bar plot illustrating the haplotype of each offspring.

The inputs of the program are as follows: a VCF file containing the variants in the given region, the chromosome name, and the start and end positions of the given region.

The program works by the following steps:

1- Iterating through the given region and separating the variants (one parent is HET and the other is HOM) into two tables.
2- Iterating through each row in each table separately, the program checks for the grouping (offspring that are HET are in the first group, and those that are HOM are in another group) and creates a dictionary for the grouping. The key is the group, and the item is the number of occurrences of this discovered grouping.
3- The reported grouping at each haplotype is the grouping that shows the greatest abundance, i.e., the key that has the highest number of its items.
4- Optionally, a bar plot is generated.

Figure S6 shows the mother haplotype phasing in the offspring, and the phasing of the father haplotype follows the same principle.

In one table, we classify all the variants that are HET in the mother and HOM in the father. We iterate through the rows and define the offspring whose genotypes are similar to that of the mother. Next, we define the grouping and count the incidents.

In this case, the first four variants in offspring 1, 2, and 3 are grouped with the mother, and this represents the most common grouping; therefore, this is the grouping (A) that will be reported in the final phasing of the mother's haplotype.

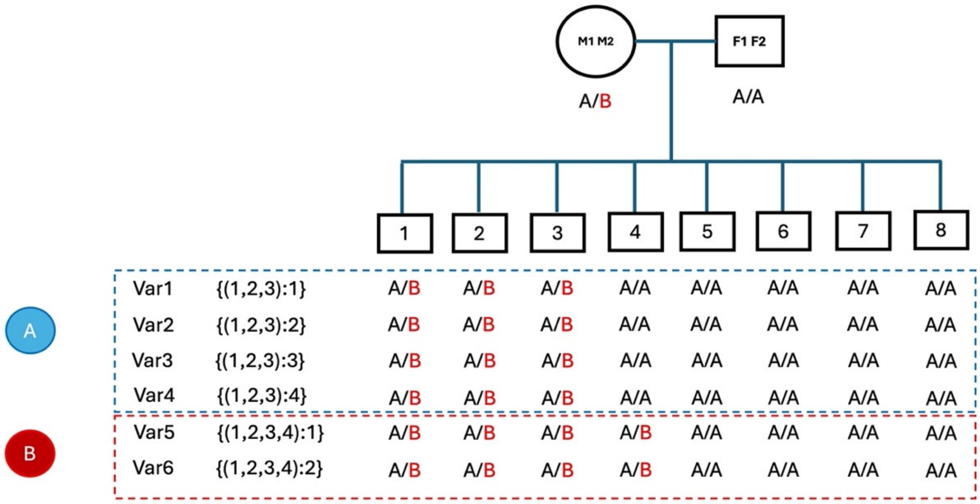

Figure S6: Haplotype block phasing in the offspring on the basis of the heterozygous variants in the parents found in the gene or in the extended region if necessary. The heterozygous variants (A/B) in each offspring are shown, and the incidences that follow the same pattern (indicating the same block or stretches of homozygosity) are counted. Option A will be chosen as the phasing block of the mother haplotype since it finds four variants to conform the pattern against two variants in the B phasing option.
