# Supplementary material for: A New Family-Based Approach for Detecting Allele-Specific Expression and for Mapping Possible eQTLs
Source: Animals (Basel). 2025 Sep 22;15(18):2766. doi: 10.3390/ani15182766 (PMC12466419; doi:10.3390/ani15182766)
Supplement: Supplementary file 1 [file animals-15-02766-s001.zip › Figure S7.pdf]

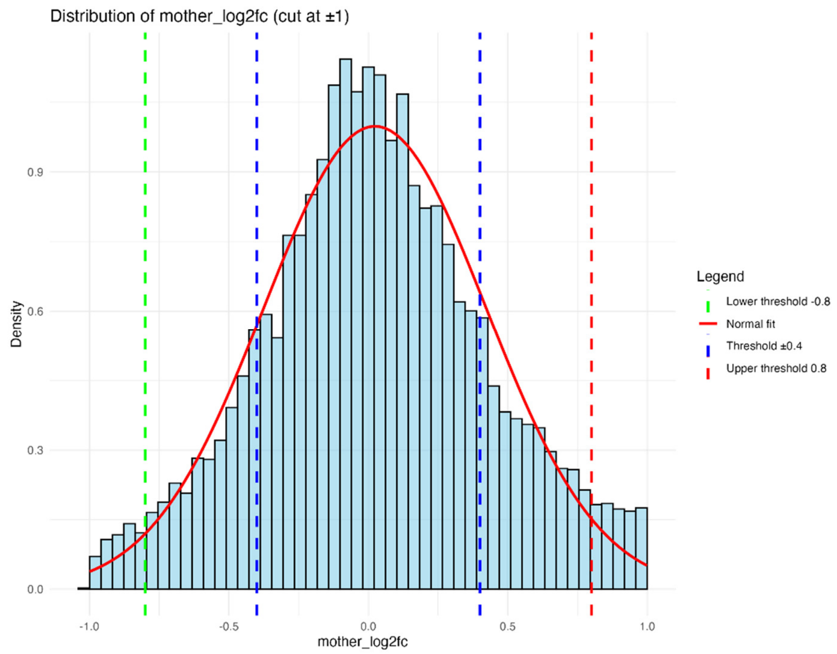

Figure S7: Distribution of mother Log2FC. The plot presents the mother's Log2FC distribution modeled as a standard normal distribution with the cutoff [-1, +1], as the father is the baseline for comparison. The y-axis shows the density of the Log2FC values in the mother. Moderate expression is defined as z-scores between −0.4 and +0.4 (Between blue dashed lines). High expression ≥ 0.8 (Red dashed line cutoff) and low expression ≤ −0.8 (Green dashed line). These thresholds are the upper and lower 20% quantiles, replacing the conventional 25% quartile cutoffs for a sharper boundries.

The Log2FC values for the mother follow a normal distribution. The mother was used as a reference in the comparison to determine threshold values for classifying gene expression patterns. We focused on cases where at least one individual had a |Log2FC| greater than 1, which is considered significant, and we created the plot for the range −1 to +1 to highlight this region.

This allowed us to categorize gene expression into biologically significant groups. We based this on the distribution of normalized values following a standard normal distribution. While the classification of H_L and L_H, was simply based on the |Log2FC| > 1, this distribution was especially important for classifying the cases that involve M in the mother. Genes with Log2FC values around the mean ($\mu \approx 0$) were classified as moderately expressed (M). Conventionally, quartile-based classification assigns the lowest 25% (1st quartile) and highest 25% (4th quartile) as low and high expression, respectively, based on Log2FC values. To reduce potential false positives and improve reliability at the extremes of expression (i.e., high and low), we applied a more stringent 20% cutoff at each end of the distribution. This cutoff corresponds to ±0.8 standard deviations.
On the other hand, to reduce false negatives, the middle zone had to be widened. Consequently, genes with Log2FC between −0.4 and +0.4 were considered moderately expressed, those with values less than or equal to −0.8 were classified as lowly expressed, and those with values greater than or equal to +0.8 as highly expressed. Genes outside these ranges, thus falling into the transition zone, are not included in the analysis. This categorization method balances sensitivity and specificity by leveraging the shape of the distribution.
